# Sea Cucumber Peptides Improved the Mitochondrial Capacity of Mice: A Potential Mechanism to Enhance Gluconeogenesis and Fat Catabolism during Exercise for Improved Antifatigue Property

**DOI:** 10.1155/2020/4604387

**Published:** 2020-06-26

**Authors:** Yihao Yu, Guoqing Wu, Yuge Jiang, Bowen Li, Chuanxing Feng, Yueting Ge, Han Le, Liang Jiang, Huiping Liu, Yonghui Shi, Guowei Le

**Affiliations:** ^1^State Key Laboratory of Food Science and Technology, Jiangnan University, Wuxi, Jiangsu 214122, China; ^2^Center for Food Nutrition and Functional Food Engineering, School of Food Science and Technology, Jiangnan University, Wuxi, Jiangsu 214122, China; ^3^Era Biotechnology (Shenzhen) Co., Ltd., Shenzhen 518000, China

## Abstract

Sea cucumber promotes multifaceted health benefits. However, the mechanisms of sea cucumber peptides (Scp) regulating the antifatigue capacity is still unknown. The present study is aimed at further elucidating the effects and mechanisms of Scp on the antifatigue capacity of mice. At first, C57BL/6J mice were assigned into four groups named Con, L-Scp, M-Scp, and H-Scp and received diets containing Scp (0%, 0.15%, 0.3%, and 0.5%, respectively) for continuous 30 days. On the 21th day, a fore grip test was conducted on mice. On the 25th day, a rotating rod test was conducted on mice. On the 30th day, the quantities of glycogen and mitochondrial DNA (mtDNA) were determined in 8 random mice and another 8 mice were forced to swim for 1 hour before slaughter for detecting biochemical indicators. It was observed that the Scp groups significantly prolonged the running time in rotarod, increased forelimb grip strength, improved lactic acid (LD) and urea nitrogen (BUN) levels in the serum, decreased lactic dehydrogenase (LDH) and glutamic oxalacetic transaminase (GOT) activities in the serum, increased blood glucose (BG) and glycogen (GN) levels in the liver and skeletal muscle after swimming, increased the activity of Na^+^-K^+^-ATPase and Ca^2+^-Mg^2+^-ATPase in the skeletal muscle and heart, and improved antioxidant capacity. Furthermore, Scp treatment significantly elevated the mRNA and protein relative levels of power-sensitive factors, lipid catabolism, and mitochondrial biogenesis and significantly upregulated mRNA levels of gluconeogenesis. Besides, mtDNA before the swimming test was increased in the three Scp groups. These results show that Scp treatment has antifatigue capacity. Furthermore, these results suggest that improved energy regulation and antioxidant capacity may be the result of improved mitochondrial function.

## 1. Introduction

Fatigue is often set in the certain peak of mental or physical status required to sustain a given task, indicating a temporary decrease in work performance and a sign of several illnesses [1]. It has been well established that a low level of exercise capacity is associated with cardiovascular disease, mass loss, and atrophy [2]. With the rapid development of modern society, many people are unable to achieve the sports needed to maintain health for the several reasons: (1) age, (2) injury, (3) illness, (4) work, or (5) social commitments. Therefore, the development of therapeutic mimetics that induce sports benefits could provide a wide range of benefits to the medical community [3].

There are many theories about the mechanisms of physical fatigue, including the exhaustion theory, the clogging theory, the radical theory, and the protective inhibition theory [4]. The exhaustion theory suggests that low utilization and depletion of endogenous fuel such as glucose and liver glycogen lead to fatigue [5]. In addition, the occurrence of fatigue is accelerated by the imbalance of metabolic utilization accompanied by the accumulation of metabolites such as lactic acid and urea nitrogen. One of the reasons for antifatigue is the body's ability to maintain energy homeostasis and reduce energy consumption [6]. To cope with the rapid neurological fatigue due to glycogen and glucose depletion, adaptions were done immediately following exercise response rapid carbohydrate consumption through the way of increased fat using efficiency and gluconeogenesis, which significant in improving endurance performance [7]. Generally, high-energy regulation flexibility is a sign of high endurance performance and exercise adaption. AMPK signaling is responsive to changes in AMP/ATP turnover, and exercise under energy-restricted circumstances may further accentuate AMPK signaling and enhance metabolically beneficial adaptations [8, 9]. Moreover, AICAR (5-amino-1-*β*-d-ribofuranosyl-imidazole-4-carboxamide), a chemical AMPK activator, has been shown to increase endurance capacity in animal studies [10]. The ability of the heart to pump blood and skeletal muscles to move and the role of the liver as an energy hub have been increasingly valued by researchers. In the present study, we explored gluconeogenic pathways; fat catabolism in the heart, liver, and skeletal muscle; and the expression of energy-sensing components to further study the antifatigue mechanism of Scp.

On the other hand, enhanced mitochondrial functions improved the balance of energy metabolism. Mitochondria are the molecular layer of energy metabolism maintenance which transforms metabolism intermediates, such as pyruvate, fatty acids, and amino acids, into reduced energetic equivalents through *β*-oxidation and the TCA cycle [11]. As such, mitochondria are the site for the generation of ATP and the primary organelle that maintains the balance between ATP generation and hydrolysis. Furthermore, mitochondrial adaptions decide exercise adaptions. High mitochondrial content and quality strongly associated with muscle oxidative capacity which determines the output of ATP and power and endurance performance [12, 13]. However, few studies have been conducted to explain the mitochondrial capacity of the skeletal muscle and myocardium in an exercise fatigue model and contributed the improved ability to regulate energy metabolism during exercise to the enhanced function of the mitochondria, and increased glycogen storage also blurs the role of mitochondria in endurance performance. In a previous study, the improved activity of ATPase and glycemic balance and glycogen storage were detected after forced swimming test with the assistance of Scp administration without exploring the feasible mechanism [14].

Sea cucumbers have long been used for folk medicine and food in China and other countries in Asia [15]. They have an impressive character of valuable nutrients including triterpene glycosides, chondroitin, polysaccharides, phenolics, and essential fatty acid which are recognized as the origin of unique biological and pharmacological functions of sea cucumbers including antimicrobial, antioxidant, immunoregulation, and antihypertension [16]. Studies and ancient books and records show that taking sea cucumber has an obvious antifatigue effect [17]. Further studies showed that peptides hydrolyzed from the body wall of the sea cucumber enhanced the time in swimming experiment and decreased glycogen consumption and increased antioxidant capacity during exercise [18]. The protein makes up more than 70% in sea cucumber body, which is an effective source of food-borne bioactive peptides. It is well known that endogenous biologically active peptides function as a hormone-regulating energy state during various physiological states, and there were lots of studies that reported the function of exogenous peptides in maintaining energy homeostasis during prolonged exercise and delayed exhaustive time [4]. However, most of the current research on antifatigue substances were only limited to the knowledge that the substances have antifatigue functions, which is not conducive to pharmacological intervention. Up to now, there was no study researched about the effect of Scp on energy metabolism balance and regulation during exercise, which are the basis of endurance performance and antifatigue capacity. Therefore, we aim to further identify the antifatigue target of sea cucumber peptide and find the corresponding molecular evidence.

## 2. Materials and Methods

### 2.1. Preparation and Identification of Scp

#### 2.1.1. Preparation of Sea Cucumber Peptides

The Scp sample, which was derived from the body wall of S. japonicus grown in Jiangsu province in China, was provided by Jidan Biological Co. LTD (Jiangsu, China). The extraction method of protein was referred to Jin et al. with little modification [19]. Briefly, after removing the viscera of the live sea cucumber, its body wall was boiled in water for 30 min to inactivate an endogenous enzyme. Afterward, the mincing part of the body wall was soaked in 10 volumes of 0.2 M EDTA (pH 8.0) for 48 h to remove the heavy metals; and then, the EDTA solution was removed. Then, 10 volumes of 15% (*v*/*v*) butyl alcohol were added to the sediment and soaked for 48 h. The sediment was washed three times with the deionized water and then soaked in 10 volumes of 0.5 M acetic acid for 48 h. NaCl was added into the filtrate obtained by two layers of cotton cloth with the final concentration of 2.5 M. After centrifugation at 12,000 rpm for 30 min, the precipitate was collected and dissolved in a 0.5 M acetic acid solution. The resulting solution was dialyzed against the deionized water for 24 h with a change of solution every 12 h. The sea cucumber protein was obtained after lyophilizing the resulting dialysate.

The enzymatic hydrolysis method was referred to Ye et al. to get Scp. In brief, the lyophilizing powder was suspended in distilled water (50 mg/ml) and hydrolyzed with neutral protease at a ratio of enzyme-to-substrate 1 : 100 (*w*/*w*) for 4 h at 50°C and pH 7; the solution was then boiled for 10 min to inactivate the enzyme and centrifuged at 12,000 g for 15 min. Protein hydrolysates were fractionated using an ultrafiltration membrane (5 kDa) procured from Pall Corporation (New York, USA). The fraction with a molecular weight of less than 5 kDa was collected as Scp and lyophilized and stored in a desiccator for further use.

#### 2.1.2. Determination of Molecular Weight Distribution and Amino Acid Composition of SCP

The lyophilized Scp powders were put in a tube containing 8 ml 6 M HCl and then hydrolyzed in an anaerobic environment at 120°C for 22 hours. After hydrolysis, the sample was neutralized by 4.8 ml 10 M NaOH and diluted with water to 25 ml. The obtained liquid compound was filled with double filter paper. The strained sample was sent to the State Key Laboratory which used a high-performance liquid chromatograph (Agilent 1100 HPLC, Tokyo, Japan) to obtain the composition of amino acid in Jiangnan University.

Scp 100 mg powers were diluted with mobile phase to 10 ml scale in a volumetric flask, ultrasonic for 5 min, and then filtered by microporous filtration membrane after centrifugation for sample injection. The contents of the Scp sample were estimated using HPLC (Waters 1525 HPLC, American, USA) with a TSKgel 2000 SWXL at 300 mm × 7.8 mm. The mobile phase consisted of acetonitrile/water/trifluoroacetic acid (45 : 55 : 0.1, *v*/*v*/*v*) which was run at a flow rate of 0.5 ml/min. Cytochrome C (Mw 12,384 Da), aprotinin (Mw 6512 Da), bacitracin (Mw 1422 Da), and glycine-glycine-tyrosine-arginine (Mw 451 Da) and glycine-glycine-glycine (Mw 189 Da) were used as the Mw markers.

### 2.2. Animals and Experiment

#### 2.2.1. Animals

This study was received and approved by the Animal Care and Use Committee at Jiangnan University. Seven-week-old male C57BL/6J mice (*n* = 64, weighing 33 ± 2 g) were purchased from Shanghai Slack Laboratory Animals Co., Ltd. (Shanghai, China). All mice were housed in a room at a temperature of 22 ± 2°C and relative humidity of 50% ± 10% on a 12 : 12 h light-dark cycle at Jiangnan University Center for Animal Experiment (Wuxi, China). They were given free access to food and drinking water throughout the experiment. After 1 week of acclimation, all mice were randomly divided into the following four groups: (1) Con mouse group was fed with standard commercial diet according to the AIN-93M (*n* = 16); (2) L-Scp mouse group was fed low-dose Scp, (*n* = 16, 1.5 g Scp per kilogram feed); (3) M-Scp mouse group was fed medium-dose Scp (*n* = 16, 3.0 g Scp per kilogram feed); and (4) H-Scp mice group was fed high-dose Scp (*n* = 16, 3.0 g Scp per kilogram feed). The feed was purchased from Xietong Bio Inc., Jiangsu, China. Bodyweight and food intake of each mouse were weekly measured. The whole experimental period was 30 consecutive days.

#### 2.2.2. Forelimb Grip Strength Test

We performed the forelimb grip strength test according to a previous study with an appropriate modification [20]. The experimental procedures were as follows: a Grip Strength Meter (YLS-13A; Jinan Yiyan technology development Co. Ltd., China) equipped with several metal bar force transducer was used to measure the forelimb grip strength of mice. In this experiment, the mice were allowed to grasp the bar mounted on the force gauge with only their front paws and the peak pull force in grams was recorded on a digital force transducer during the process of steady grasping until losing strength. After each mouse finished, we used 75% ethanol to rinse the instrument and then dried it to ensure that the next mouse would not be affected by the previous secretion. The measurement procedure was that our technician conducted the process at a constant speed sufficiently slow to permit mice to build up a resistance against the bar, and the date was only recorded vertically in the horizontal plane. The forelimb grip strength test was performed at day 21 of the treatment, and each mouse was measured 10 times to receive an available date and then averaged them for comparison among each group.

#### 2.2.3. Rotating Rod Test

On day 24 of indicated Scp administration, three independent trainings on turning device was performed according to previous study with little modification [21]. Firstly, each mouse was put on the stationary rod, and then the turning device was accelerated at a constant speed to 15 from 0 within 1 min. After resting for 1 hour, the second adaptation experiment was conducted. Like the first one, mice were placed on a stationary wheel, but the speed was attained to 18 from 0 within 1 min. The third experiment was identical to the second one after 1 hour it finished. On day 25 of treatment, a formal experiment was conducted. There were two independent devices with 5 openings of each device to experiment. Consistent with the preliminary one, each mouse was placed on the stationary rod, and the speed was accelerated to 18 within 1 min; and then, the time taken for mice to succumb to muscle fatigue and fall off the device was recorded.

#### 2.2.4. Forced Swimming Test

On the 30the day of indicated Scp administration, 8 mice from each group, forced swimming test was performed in a total of 32 mice . This experiment began at 8 o'clock on the morning of the 30th day. Briefly, each mouse was placed individually in a swimming pool filled with water (27 ± 1°C) to a depth of 1 m according to the time arrangement. Each swimming pool cover one square meter with the total number of eight and there were no more than 4 mice per square meter. Every 3 min, we put a mouse to a swimming pool and with the 4 groups performed concurrently. After each mouse finished their swimming, we dried the mice with a towel and then the mice were slaughtered.

### 2.3. Sample Collection

After 10-11-hour fasting, the 8 mice in each group were forced to swim for 1 hour. At the end of the experiment, mice were anesthetized by pentobarbital sodium (50 mg/kg BW, i.p.). Immediately after this process, blood was drawn from the retroorbital sinus into a serum separator tube containing sodium heparin as an anticoagulant. After the process of blood extract, the mice were sacrificed by cervical dislocation. The blood sample was kept at 4°C for 30 min, and then the plasma samples were obtained by centrifugation at 3500 rpm at 4°C for 15 min. The plasma used for biochemical detection was stored at -80°C. The tissues of the liver, gastrocnemius muscle, and heart were immediately collected, weighted, washed with sterile 0.9% (*w*/*v*) NaCl solution, and then cut into three parts saved in a tube, respectively, one part for biochemical analysis, one part for real-time quantitative polymerase chain reaction (RT-qPCR), and one part for Western blot analysis. After postproposing, each tube was flash frozen in liquid nitrogen and stored at −80°C until use. After 11-12 hours of fasting, another 8 mice in each group were sacrificed by cervical dislocation before anesthesia, and only the liver, gastrocnemius muscle, and heart were extracted to measure GN and mtDNA content.

### 2.4. Regents

EDTA, Tris, NaCl, HCl, NaOH, cetonitrile, trifluoroacetic acid, cytochrome C, aprotinin, bacitracin, glycine-glycine-tyrosine–arginine, and glycine-glycine-glycine were obtained from Sinopharm Chemical Reagent Co., Ltd. (Shanghai, China). Assay kits for biochemical detection of total antioxidant capacity (TAC), glutathione peroxidase (GPX), catalase (CAT), Malondialdehyde (MDA), BUN, LD, GN, LDH, Na^+^k^+^ATPase, Ca^2+^Mg^2+^ ATPase, and GOT were purchased from the Nanjing Jiancheng Bioengineering Institute (Nanjing, China). The bicinchoninic acid (BCA) assay kit was purchased from Thermo Fisher Scientific (Waltham, MA, USA). TRIzol reagent was purchased from Vazyme Biotech Co., Ltd. (Suzhou, China). RIPA lysis buffer and 5x loading buffer were purchased from Beyotime Institute of Biotechnology (Shanghai, China). Gene primers were designed and synthesized by Generay Biotech Co., Ltd. (Shanghai, China). Primary antibodies against GAPDH, p-AMPK, AMPK, SIRT1, PGC1*α*, TFAM, PPAR*σ*, and PPAR*α* were purchased from Abcam Co., Ltd. (Shanghai, China). Secondary antibody was supplied by LI-COR, Inc. (Lincoln, Nebraska, USA).

### 2.5. mtDNA Copy Number Analysis

Total DNA was extracted from tissue samples using the Cell/Tissue DNA Isolation Kit (Generay Bio Inc, Shanghai, China). Concentration and purity of the extracted DNA in each sample were measured using NanoDrop ND-2000 spectrophotometer (Thermo, Waltham, MA, USA). Measurement principle was reported by Gonzalez Hunt et al. [22] The relative quantification of mt-ND2 (mitochondrial DNA, mtDNA) and control n*β*2 M (nuclear DNA, nDNA) was performed.

### 2.6. Total RNA Extraction and Quantitative RT PCR (qRT-PCR)

Total RNA of the liver, gastrocnemius muscle, and heart were done by TRIzol method according to the manufacturer's protocol (Applied Biosystems, Foster City, CA, USA). The purity and amount of the extracted RNA in each sample were evaluated by NanoDrop Spectrophotometer (ND2000, Thermo, Waltham, MA, USA). Subsequently, reverse transcription was conducted by mRNA reverse transcription kits (Vazyme. Suzhou, China). An SYRB green-based qRT-PCR kit was used to analyze mRNA expression levels according to the manufacturer's instructions of the 7900HT instrument (Applied Biosystems, Forster, CA, USA). The primer sequences are shown in Table 1. The gene expression data were normalized by comparing to the relative mRNA expression level of *β*-actin.

### 2.7. Western Blot Analysis

Frozen tissues of the liver, skeletal muscle, and heart were lysed with ice-cold radioimmunoprecipitation assay (RIPA) lysis buffer including a protease inhibitor cocktail. The mixtures were centrifuged at 12,000 rpm at 4°C for 5 min. Afterward, the collected supernatants were assayed with a BCA kit to test the content of protein. When the same concentrations of protein levels were obtained, 5x loading buffer was added. The mixture was reacted at 95°C for 15 min. Then, it was separated on 10% SDS-PAGE and transferred to a nitrocellulose membrane (Bio-Rad). The membrane was incubated with primary antibodies of GAPDH, p-AMPK, AMPK, SIRT1, PGC1*α*, TFAM, PPAR*α*, and PPAR*σ* overnight at 4°C and then incubated for 1 h with horseradish peroxidase-conjugated served as secondary antibodies. Respective proteins were analyzed by using ECL Plus (Amersham Biosciences). The resulting blots were visualized and quantified by using Image-Pro Plus software (Tannon 5200, Shanghai, China). The protein expression was relatively quantified by GAPDH.

### 2.8. Statistical Analysis

All data are represented as the mean ± standard error of the mean (SEM) values. The result was analyzed by one-way ANOVA, followed by Tukey's test. Before each ANOVA, we conducted a normal distribution test. When equal variance could not be assumed, we used one-way ANOVA with Tamhane's T2 post hoc test to evaluate the statistical significance. When a *p* value less than 0.05, the statistical significance was obtained. All data were statistically analyzed by software SPSS 17.0 (SPSS Inc., Chicago, USA).

## 3. Results

### 3.1. Molecular Weight Distribution and Amino Acid Composition of Scp

**Figure 1:**
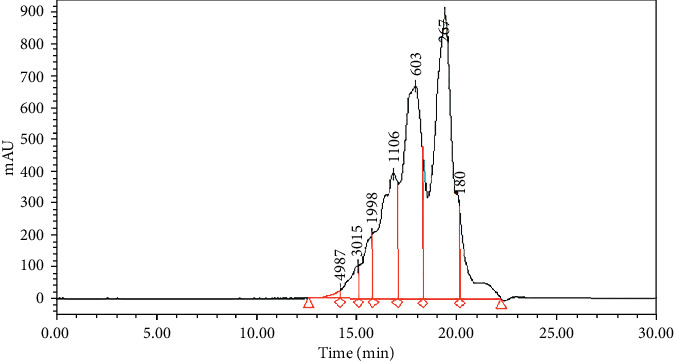
The molecular weight distribution of SCP.

As shown in Figure 1, the Scp consists of a series of oligopeptides characteristic of a relative molecular weight less than 2000 Da that was 92.99%. There were 71.29% of peptides distributed in the molecular range from 1106 Da to 267 Da, and the detail was that 1000 Da to 500 Da was 15.88% and 500 Da to 180 Da was 42.07%.

The amino acid composition of Scp is shown in Table 2. The amino acid content of Scp by acid hydrolysis extraction accounted for 74.13% of the total weight of Scp, which was consistent with the results of Ye et al. [18, 19] The amino acid content of Scp is up to 80.5%, but it contains 7% tryptophan. The highest content amino acids in Scp were aspartic acid, glutamic acid, glycine, arginine, and proline.

### 3.2. Effects of Scp on Body Weight, Feed Intake, and Heart and Skeletal Muscle Mass

**Figure 2:**
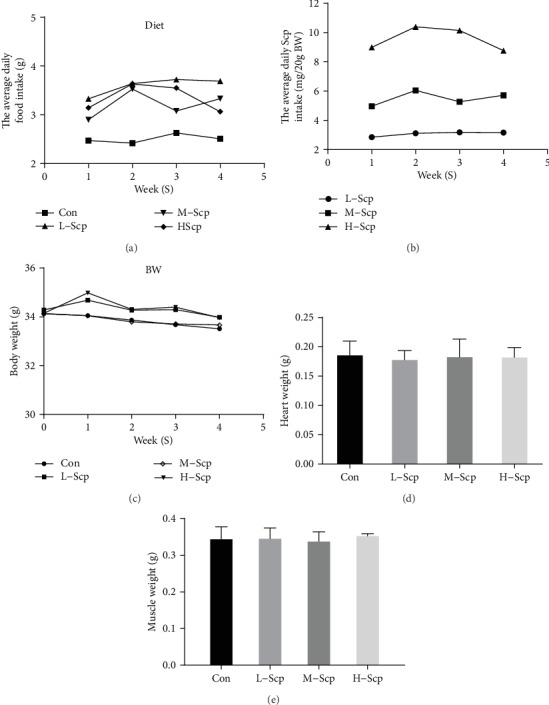
Effects of Scp on body weight, exercise-related organ weight, and feed intake. (a) Bodyweight, (b) daily feed intake, (c) the actual Scp daily intake, (d) heart weight, and (e) skeletal muscle weight. Con: the control group; L-Scp: Scp low-dose group; M-Scp: Scp moderate-dose group; H-Scp: Scp high-dose group. All data are presented as mean ± SEM (*n* = 16).

The three Scp groups had significantly higher feed intake than the Con mice throughout this experiment (*p* < 0.05), and there was no difference in feed intake observed among the three Scp groups (Figure 2(a)). According to the diet data, we calculated the actual intake of Scp and the result was that the L-Scp mice had an average of 3 mg per 20 g bodyweight daily, the M-Scp had 5.8 mg, and the H-Scp had 9.5 mg (Figure 2(b)). However, there was no trend that manifested that any difference in body weight (BW) existed among all groups (Figure 2(c)), thereby indicating that Scp administration should exert metabolic influence on mice causing the effect of adephagia and the bodyweight balance. The organ weight of the heart and skeletal muscle also shown no difference among all groups and the results suggested that, in the Scp treatment, improved antifatigue capacity was not through increased exercise-related visceral mass in adult mice in a month (Figures 2(d) and 2(e)).

### 3.3. Effects of Scp on Forelimb Grip Strength and Rotating Rod Test

**Figure 3:**
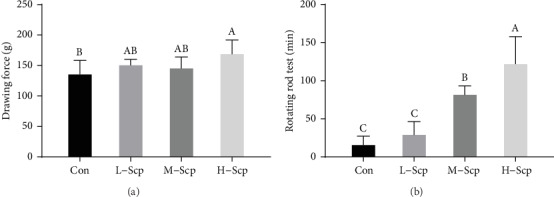
Effects of Scp on the forelimb grip strength (a) and rotarod performance (b). All data are presented as mean ± SEM (*n* = 16). Con: the control group; L-Scp: Scp low-dose group; M-Scp: Scp moderate-dose group; H-Scp: Scp high-dose group. The means marked with superscript letters are significantly different relative to others.

The grip strength test is designed to evaluate the functions and strength of muscle. As shown in Figure 3(a), the values of an average forelimb grip strength in the Con, L-Scp, M-Scp, and H-Scp mice were 137.2 g, 152.1 g, 147 g, and 170.3 g, respectively. The grip strength of the H-Scp mice was significantly higher than that of the Con mice (*p* < 0.05).

The rotarod performance test is a test usually evaluating motor coordination and endurance according to the length of time that a given animal stays which are determined by energy metabolism and willingness to exercise. As shown in Figure 3(b), the M-Scp and H-Scp mice markedly increased the length of the staying time in a dose-dependent manner compared to the Con mice (*p* < 0.05). The lengths of time in L-Scp, M-Scp, and H-Scp mice were 1.42-, 3.07- and 4.56-fold compared to that of in Con mice, indicating that Scp treatment had a significant antifatigue capacity on mice.

### 3.4. Effects of Scp on Fatigue-Related Biochemical Indexes

**Figure 4:**
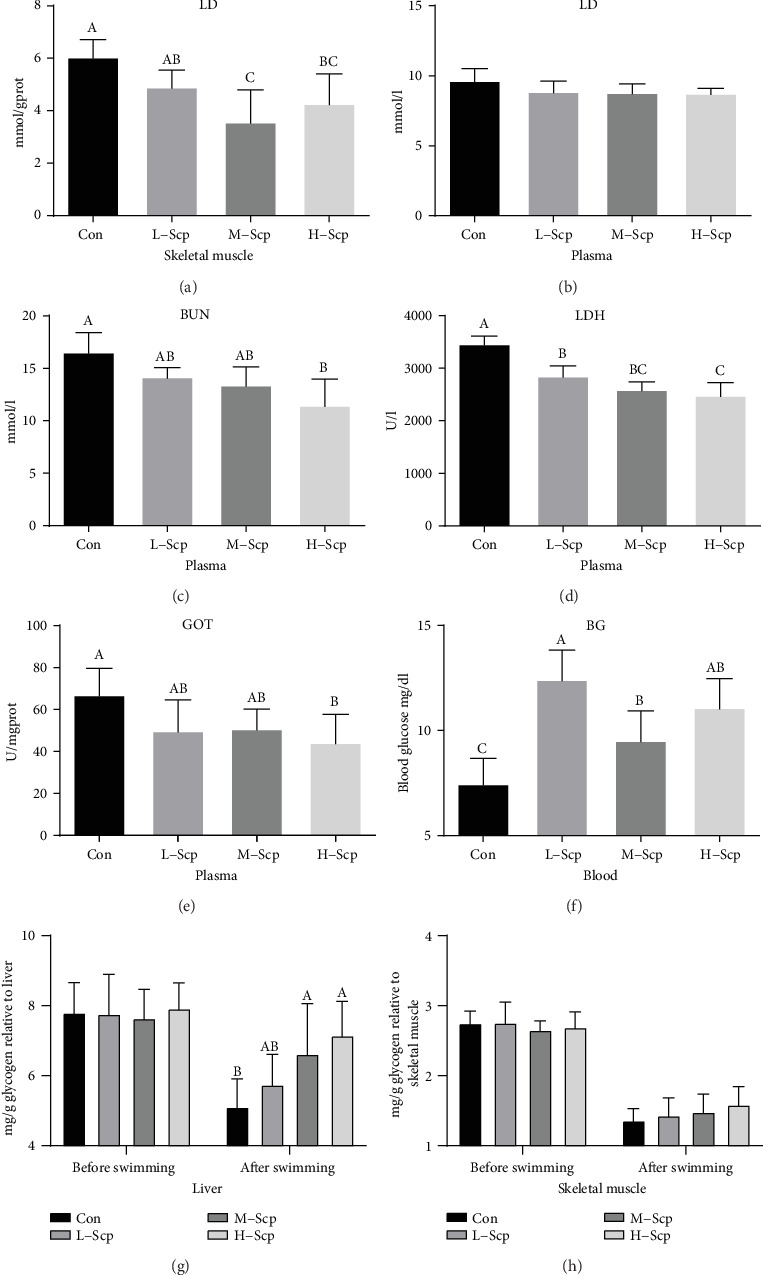
Effects of Scp on fatigue-related biochemical indicators. (a) Muscular LD, (b) plasma LD, (c) plasma BUN, (d) plasma LDH, (e) plasma GOT, (f) BG, (g) liver glycogen before and after exercise, and (h) muscle glycogen before and after exercise. Con: the control group; L-Scp: Scp low-dose group; M-Scp: Scp moderate-dose group; H-Scp: Scp high-dose group. All data are presented as mean ± SEM (*n* = 8). The means marked with superscript letters are significantly different relative to others.

Firstly, we measured the biochemical indicators related to fatigue and tissue damage after the completion of the isochronous exercise. LD is considered to indicate insufficient oxygen supply in working muscles. High levels of LD decrease pH value and then alter intracellular metabolism homeostasis, reducing contractility in the muscle [4]. As shown in Figure 4(a), the M-Scp and H-Scp mice significantly decreased the LD levels in the skeletal muscle than the Con mice (*p* < 0.05). However, there was no difference in the amount of LD among all groups in the plasma (Figure 4(b)). BUN, a protein degradation product, is a sensitive index to assess body endurance for only when carbohydrate and fat cannot meet the energy supply where there will be a situation of accelerated protein oxidation [4]. In the plasma, the H-Scp mice significantly decreased the BUN quantities (Figure 4(c)) compared to the Con mice (*p* < 0.05), indicating that the energy metabolism homeostasis was improved with the help of Scp treatment. LDH catalyzes the conversion of LD to pyruvate and back existing in the muscular tissue. On plasma tests, an elevated level of LDH usually indicates muscle damage [4]. The Scp group mice significantly decreased the quantities of LDH in plasma (Figure 4(d)) compared to that in the Con in a dose-dependent manner (*p* < 0.05). Besides, GOT mainly exists in the heart and catalyzes amino acid deamination, and high levels of GOT means myocardial damage [4]. Scp treatment dose dependently decreased the activity of GOT in plasma (Figure 4(e)) and H-Scp mice had a significant difference (*p* < 0.05), indicating that Scp treatment alleviated myocardial damage. It is well known that a decrease in blood sugar and glycogen due to prolonged exercise causes fatigue. As shown in Figure 4(f), the three Scp groups significantly increased the levels of blood sugar than the Con group (*p* < 0.05). Furthermore, as shown in Figure 4(g), there was no significant difference in glycogen content in the liver muscle before exercise, and the H-Scp and M-Scp mice significantly increased the content of liver glycogen compared to the Con mice after prolonged exercise (*p* < 0.05). Meanwhile, Scp treatment exerted an increasing trend on the content of muscle glycogen in a dose-dependent manner compared to the Con group, but there was no statistically significant difference among all groups (Figure 4(h)).

### 3.5. Effects of Scp on Gluconeogenesis and Fat Catabolism

**Figure 5:**
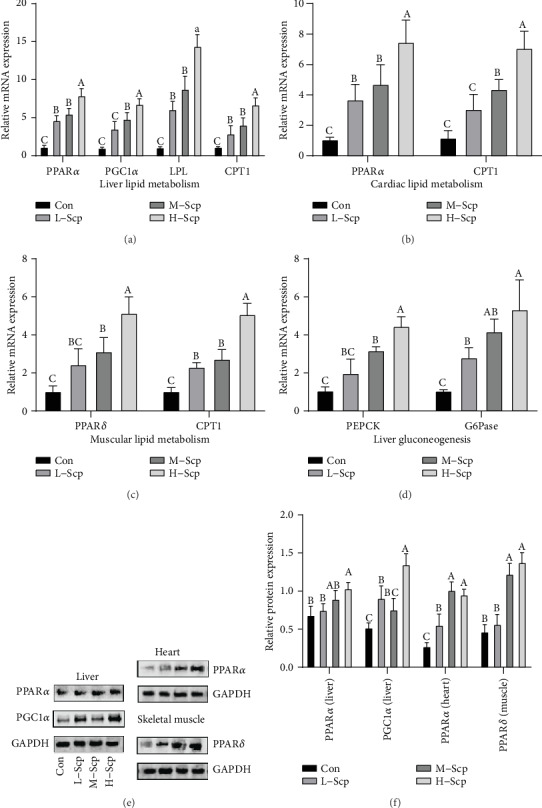
Effects of Scp on the levels of mRNA expression and protein expression of lipid metabolism and glycometabolism. (a–d) mRNA expression levels: (a) hepatic lipid metabolism, (b) heart lipid metabolism, (c) muscle lipid metabolism, and (d) liver gluconeogenesis. Protein levels of the lipid metabolism and gluconeogenesis regulatory elements: (e) the western band and (f) relative protein expression levels of PGC1*α* and PPAR*α* in the liver, PPAR*α* in the heart, and PPAR*δ* in the muscle. Con: the control group; L-Scp: Scp low-dose group; M-Scp: Scp moderate-dose group; H-Scp: Scp high-dose group. All data are presented as mean ± SEM (*n* = 8). The means marked withsuperscript letters are significantly different relative to others.

Systematic increase in lipid metabolism and gluconeogenesis delays glycogen consumption. To validate global lipid metabolism, we measured the expression of major regulatory elements of lipid metabolism and rate-limiting enzymes in the liver, skeletal muscle, and heart. In the liver, peroxisome proliferator-activated receptor alpha (PPAR*α*) is the primary regulator in triglyceride metabolism including the charge of mitochondrial fatty acid *β*-oxidation, absorption, and transport of fatty acids and apolipoproteins related to lipoprotein lipase (LPL) activation. Besides, peroxisome proliferator-activated receptor gamma coactivator 1-alpha (PGC1*α*) promotes the expression of PPAR*α* [23]. The results (Figure 5(a)) show that the mRNA expression levels of PGC1*α* and PPAR*α* were significantly higher in the three Scp groups in the liver compared to the Con group (*p* < 0.05). Meanwhile, protein expression levels of PPAR*α* and PGC1*α* (Figures 5(e) and 5(f)) were significantly higher in the M-Scp and H-Scp mice compared to the Con mice (*p* < 0.05). Furthermore, LPL, a rate-limiting gene for fatty acid transport, and carnitine palmitoyltransferase 1 (CPT1), a rate-limiting enzyme in fatty acid *β*-oxidation [24], were both significantly upregulated in the all Scp groups than those in the Con group in a dose-dependent manner (Figure 5(a)) (*p* < 0.05).

The heart responsible for blood pumping is powered mainly by the oxidation of fatty acids, and PPAR*α* is a controlling factor mainly regulating fatty acid catabolism in the heart [25]. Compared to the Con group, all the Scp groups significantly upregulated the mRNA expression levels of PPAR*α* (*p* < 0.05) and the M-Scp and H-Scp groups significantly upregulated mRNA levels of CPT1 (*p* < 0.05) (Figure 5(b)). Also, protein expression levels of PPAR*α* were significantly higher in the M-Scp and H-Scp mice compared to the Con mice (Figures 5(e) and 5(f)) (*p* < 0.05).

Besides, peroxisome proliferator-activated receptor delta (PPAR*δ*) is the major lipid metabolism hub in the muscle. The mRNA and protein expression levels of PPAR*δ* in the M-Scp and H-Scp mice were significantly higher than those in the Con mice (*p* < 0.05), and mRNA levels of CPT1 were significantly upregulated in the all Scp groups compared to the Con group (*p* < 0.05) (Figure 5(c)). Also, protein expression levels of PPAR*δ* were significantly higher in the M-Scp and H-Scp mice compared to the Con mice (Figure 5(e) and 5(f)) (*p* < 0.05).

On the other hand, in terms of gluconeogenic rate-limiting steps, the mRNA expression levels of phosphoenolpyruvate carboxykinase (PEPCK) and glucose 6-phosphate (G6Pase) were significantly upregulated in the H-Scp and M-Scp mice compared to the Con mice and the increased levels were also augmented with the Scp intake increased (Figure 5(d)) (*p* < 0.05).

### 3.6. Effects of Scp on AMPK and SIRT1

**Figure 6:**
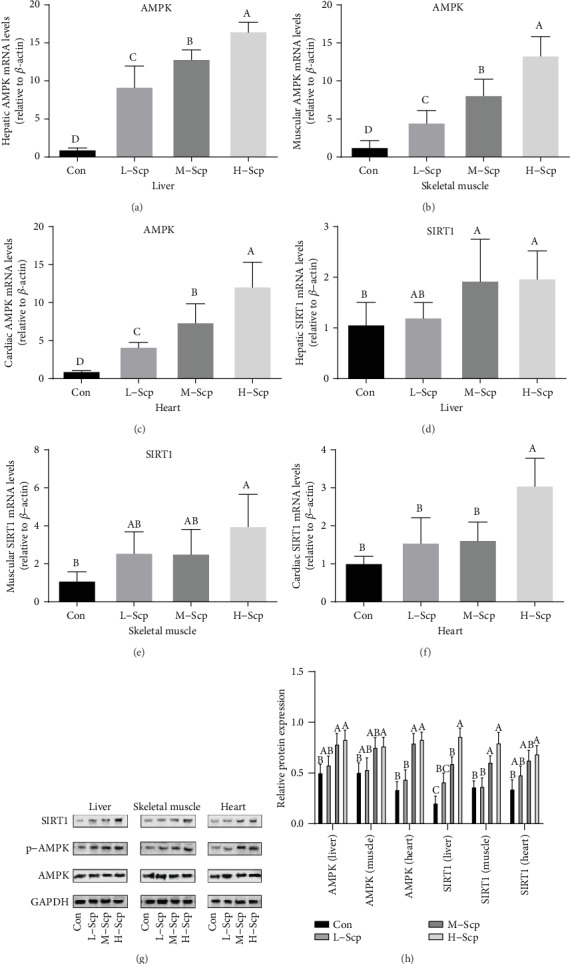
Effects of Scp on cell energy sensors. (a–f) mRNA expression levels: (a) liver AMPK, (b) muscle AMPK, (c) heart AMPK, (d) liver SIRT1, (e) muscle SIRT1, and (f) heart SIRT1. (g) pAMPK, AMPK, and SIRT1 Western blotting bands in the liver, skeletal muscle, and heart. (h) Relative protein expression levels of AMPK and SIRT1 in the liver, skeletal muscle, and heart. Con: the control group; L-Scp: Scp low-dose group; M-Scp: Scp moderate-dose group; H-Scp: Scp high-dose group. All data are presented as mean ± SEM (*n* = 8). The means marked with superscript letters are significantly different relative to others.

AMP-activated protein kinase (AMPK) and NAD-dependent deacetylase sirtuin-1 (SIRT1) are important global energy sensors in cells. The mRNA expression levels of AMPK and SIRT1 were shown in Figure 6. Compared to the Con mice, the AMPK mRNA expression levels of all Scp groups mice were significantly elevated in a dose-dependent manner in the liver, skeletal muscle, and heart (*p* < 0.05). Meanwhile, the SIRT1 mRNA expression levels of the H-Scp mice in the liver, skeletal muscle, and heart were significantly higher than those of the Con mice (*p* < 0.05). The SIRT1 mRNA expression levels of the M-Scp mice in the liver were significantly upregulated than those of the Con mice (*p* < 0.05). On the other hand, the degree of AMPK protein phosphorylation was significantly increased in the liver and heart of the M-Scp and H-Scp mice and in the skeletal muscle of the H-Scp mice compared to the Con mice (*p* < 0.05). The relative protein levels of SIRT1 of the M-Scp and H-Scp mice in the liver and skeletal muscle and the H-Scp mice in the heart were significantly upregulated than the Con mice (*p* < 0.05).

### 3.7. Effects of Scp on Mitochondrial Biogenesis and Adaptions

**Figure 7:**
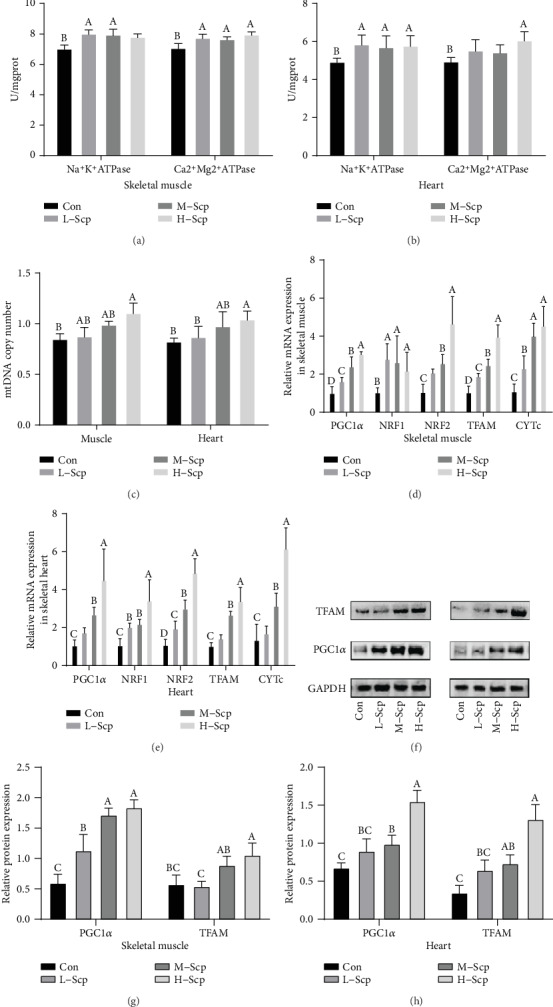
Effects of Scp on the mitochondrial biogenesis and adaptions. (a, b) ATPase activity, the activity of Na^+^K^+^ATPase and Ca^2+^Mg^2+^ATPase in muscle (a) and heart (b). (c) mtDNA content. (d) mRNA expression levels regarding mitochondrial biogenesis and adaption in skeletal muscle. (e) mRNA expression levels regarding mitochondrial biogenesis and adaption in the heart. (f) PGC1*α* and TFAM Western blotting bands in the skeletal muscle and heart. (g) relative protein expression levels of PGC1*α* and TFAM in skeletal muscle. (h) Relative protein expression levels of PGC1*α* TFAM in heart. Con: the control group; L-Scp: Scp low-dose group; M-Scp: Scp moderate-dose group; H-Scp: Scp high-dose group. All data are presented as mean ± SEM (*n* = 8). The means marked with superscript letters are significantly different relative to others.

**Figure 8:**
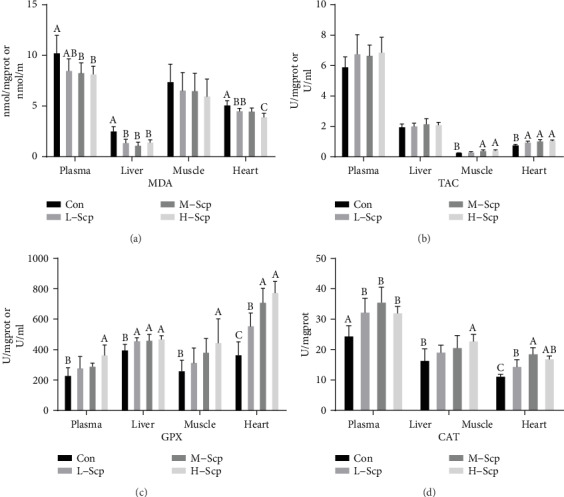
Effects of Scp on oxidative stress and antioxidant-related biochemical indicators. (a) MDA levels in plasma and tissues. (b) TAC levels in plasma and tissues. (c) GPX activities in plasma and tissues. (d) CAT activities in tissues. All data are presented as mean ± SEM (*n* = 8). The means marked with superscript letters are significantly different relative to others.

Firstly, the ability of synthesis and decomposition of ATP was measured, reflecting the turnover of ATP and mitochondrial capacity. ATPase is located in membranes that catalyze the decomposition of ATP into ADP. As shown in Figures 7(a) and 8(b), Scp administration showed an increasing trend in ATPase levels. In the skeletal muscle, the activity of Na^+^K^+^ATPase in the M-Scp and L-Scp mice was significantly higher (*p* < 0.05) than that in the Con mice (Figure 7(a)). Ca^2+^Mg^2+^ATPase activity was significantly increased in the all Scp groups compared to that in the Con group (*p* < 0.05). In the heart, Na^+^K^+^ATPase activity was significantly improved in the three Scp groups compared to that in the Con group (*p* < 0.05). The H-Scp mice significantly increased the activity of Ca2+Mg2+ATPase compared to the Con mice (*p* < 0.05). Meanwhile, CYTc, a member in the electron transport chain, is involved in the synthesis of ATP, and its high activity means a greater ability to resperation. Compared to the Con mice, the CYTc mRNA levels of L-Scp, M-Scp, and H-Scp mice are significantly upregulated in the skeletal muscle (Figure 7(d)) (*p* < 0.05), and the CYTc mRNA levels of the M-Scp and H-Scp mice are significantly upregulated in the heart (Figure 7(e)) (*p* < 0.05). The increased turnover of ATP indicated the improvement of mitochondrial function.

Before swimming, the mtDNA of the heart and skeletal muscle was measured. mtDNA content is an important characterization of mitochondrial overall capacity. mtDNA associates with the ability to convert food energy into usable cellular energy. The decline of mtDNA with age is also a direct cause of skeletal muscle atrophy and muscle strength decline [26]. As shown in Figure 7(c), the H-Scp mice significantly increased mtDNA levels compared to the Con mice in the skeletal muscle and heart (*p* < 0.05).

After swimming, mitochondrial biogenesis and mitochondrial adaptation indicators were measured. Both mitochondrial and nuclear genes code the coordination of proteins determining the process of mitochondrial biogenesis. PGC1*α* serves as a major regulator of mitochondria in the cardiac and skeletal muscles. Activated PGC1*α* binds and coregulates with different transcription factors, including NRF1 and NRF2, and then further activates TFAM which regulates mitochondrial DNA transcription, thus initiating the coordinated expression in mitochondrial proteins [12]. As shown in Figures 7(d) and 7(e), in the skeletal muscle, the three Scp groups significantly increased the gene expression levels of PGC1*α*, NRF2, NRF1, and TFAM in a dose-dependent manner compared to the Con group (*p* < 0.05). In the heart, NRF2 gene expression levels were increased dose dependently in all Scp groups compared to those in the Con group (*p* < 0.05). The H-Scp and M-Scp mice significantly increased the expression mRNA levels of PGC1*α*, TFAM, and NRF1 than the Con mice in a dose-dependent manner (*p* < 0.05). The same with the mRNA expression levels, protein levels of PGC1*α* were significantly upregulated in the M-Scp and H-Scp mice of the skeletal muscle and heart compared to those in the Con mice (Figures 7(g) and 7(h)) (*p* < 0.05). Meanwhile, protein levels of TFAM were significantly upregulated in the skeletal muscle of the M-Scp and H-Scp mice and in the heart of all Scp groups compared to that in the Con mice (Figure 7(h)) (*p* < 0.05).

### 3.8. Scp Relieved Oxidative Stress after Intense and Prolonged Exercise

The plasma, liver, skeletal muscle, and heart oxidative stress and oxidation resistance were detected. As shown in Figure 8(a), the H-Scp, M-Scp, and L-Scp mice significantly decreased the content of malondialdehyde (MDA), an oxidative stress marker, in the plasma, liver, and heart compared to that in the Con mice (*p* < 0.05) but there was no statistically significant difference in the muscle among all groups. Furthermore, total antioxidant ability (TAC) in the tissue and plasma also presented an increasing trend in the three Scp groups compared to the Con group, and the H-Scp and M-Scp mice in the skeletal muscle and heart have a significant difference compared to the Con mice (Figure 8(b)) (*p* < 0.05). Moreover, as shown in Figure 8(c), Scp groups significantly increased the activity of GPX in the plasma of H-Scp (*p* < 0.05); in the liver of L-scp (*p* < 0.05), M-Scp, and H-Scp (*p* < 0.05); in the muscle of H-Scp; and in the heart of L-Scp, M-Scp, and H-Scp (*p* < 0.05). Meanwhile, as shown in Figure 8(d), Scp groups significantly increased the activity of CAT in the liver of L-scp, M-Scp, and H-Scp (*p* < 0.05); in the muscle of H-Scp (*p* < 0.05), and in the heart of L-Scp, M-Scp, and H-Scp (*p* < 0.05).

## 4. Discussion

This study discussed the effects of Scp on improving endurance and elucidates some related antifatigue molecular mechanisms. The main finding of this research is that, besides improved endurance performance, Scp administration significantly increased fore grip strength, and after forced swimming test, elevated mitochondrial biogenesis, increased ATP turnover of formation and hydrolysis, improved the ability to fat catabolism and gluconeogenesis, increased antioxidant capacity, and decreased accumulation of metabolites including LA and BUN in mice and decreased tissue damage makers including LDH and GOT, thereby predicting the function of Scp on antifatigue. Meanwhile, the H-Scp group increased mtDNA content prior to forced swimming, consistent with increased forelimb grip strength, which may be responsible for the increased endurance performance.

Sea cucumber has long been consumed in China since ancient times and is a kind of nourishing food. For a long time, natural or artificial breeding of sea cucumbers has been used as a kind of high-quality medicinal food in terms of the efficacy from ancient writings. Modern biology, medicine, and pharmacology have found a large number of active substances in sea cucumber. This year, sea cucumber peptides extracted by enzymatic hydrolysis have effects such as antifatigue and accelerating wound healing in diabetic patient. We further elucidate the molecular mechanism of antifatigue of sea cucumber peptides and look forward to discovering other potential effects. In this study, we explored the adaptability of mice during exercise by using biochemical indexes of isochronous swimming assessment. Exercise is a way to assess and improve metabolism. It has been firmly established that a low level of exercise capacity is associated with cardiovascular disease. In skeletal muscles, a low level of exercise is highly relevant to mass loss and atrophy. After isochronous swimming, the physiological indicators associated with fatigue, including LD and BUN, were significantly decreased in the Scp group. At the same time, skeletal muscle and myocardial damage markers, LDH and GOT, were decreased, also indicating that Scp administration improved fitness in exercise.

First, we explored energy selection during exercise. The ability to select and decompose endogenous energy sources has a direct bearing on energy homeostasis during exercise. High intensity and long periods of exercise put a direct demand for breaking down carbohydrates in large quantities. Neuroglycopenia caused by the excessive consumption of blood glucose and glycogen causes a more rapid initiating of fatigue. Besides, direct demand for carbohydrate leads to the proportion of anaerobic glycolysis going up for energy supply [27]. The process wasted much energy due to the decrease in fuel utilization efficiency. Contrasting with the limited deposits of glycogen in the body, endogenous fat storage is large enough and can be treated as an unlimited power source during prolonged exercise [28]. Increased lipid utilization has been recognized as an effective means for improving endurance performance and delaying depletion in glycogen storage [7]. The catabolism of fatty acids, the heart's primary energy source as well as an important energy source, is enhanced during exercise with the help of Scp treatment which accelerated lipid metabolism and reduced glycogen consumption in the muscle. As shown in Figure 5, Scp treatment significantly improved gene and protein expressions of lipid metabolism regulatory element and rate-limiting steps in the liver, skeletal muscle, and heart, suggesting that long-time intervention of Scp was beneficial to the upregulation of lipid catabolism during exercise.

Furthermore, gluconeogenesis is essential to maintain glycemic balance. Generating glucose from noncarbohydrate substrates such as lactic acid, glycerol, and glucogenic amino acid, can be enhanced in exercise, so as to maintain blood glucose balance and slow down glycogen decomposition [29]. In the liver, the upregulated PPAR*α* and PGC1*α* which are the upstream regulation step partly explain the upregulation of the speed limit step of gluconeogen. In the three Scp groups, the upregulated expression of gluconeogenesis is consistent with the elevator blood sugar and glycogen storage. Further, gluconeogenesis has a high correlation with fat metabolism whether in the upstream regulation or metabolites. Glycerol produced by lipolysis is the direct substrate for gluconeogenesis. Meanwhile, with the increased intake of Scp, the lactic acid content in the Scp group showed an overall decreasing trend in the plasma and skeletal muscle, suggesting that gluconeogenesis may be one of the mechanisms by which Scp treatment reduces LD content in the body during the swimming test. All the results, as mentioned above, were beneficial to antifatigue for reduced LD content and maintaining blood glucose balance and glycogen storage, which benefited to reduce BUN production.

Secondly, improved ability to energy perception is beneficial to rapidly adapt to drastic changes in energy demands and decrease energy consumption for the best fuel utilization. AMPK acts to increase the rate of catabolic process and to decrease the rate of anabolic process to sustain intracellular energy homeostasis [30]. Many studies have shown a correlation between AMPK and endurance performance during exercise. Skeletal muscle repair was delayed and maximum tolerance was decreased in the AMPKa1 full-body knockout mice compared with wild-type mice [31]. Further exercise omics studies showed that AMPK has a wide range of biological functions such as calcium homeostasis and excitation contraction coupling, potassium and chloride transport, and mitochondrial respiration [32]. Since AMPK signaling is responsive to changes in AMP/ATP turnover, it is plausible that exercise under energy-restricted circumstances may further accentuate AMPK signaling and enhance metabolically beneficial adaptations [8, 9]. In the present study, gene and protein levels of AMPK wetre upregulated in skeletal muscle, heart, and liver. The upregulated AMPK and activated SIRT1 partly regulated by AMPK are consistent with the accelerated catabolic metabolism of fat for they can further promote the PPAR family to accelerate the regulation of fat catabolism. Although glucose catabolic metabolism is not explored in the present study, upregulation of lipid catabolism and gluconeogenesis is responsible for the increased endurance, which is often seen as a result of exercise training. It cannot be denied that the previous widely held view was that the activation of AMPK may hinder the process of gluconeogenesis, now there is more studies which found that AMPK does not have control over the flux of the production of sugar during motion. One example is that AMPK KO mice, 15-16 week old, did not exhibit different expressions of PEPCK and glucose production in both 8.5-9 h fasting and acute exercise [33]. Meanwhile, the upregulated gene and protein levels of PGC1*α* in the liver, as one of the regulatory units of gluconeogenesis, are also conducive to the regeneration of sugar and delay the occurrence of sugar depletion. Thus, the upregulation of AMPK and SIRT1 with the Scp treatment should be seen as the metabolic flexibility on a broader scale in time.

It is necessary to explain the improved energy homeostasis in Scp treatment for us. On the one hand, the body's inability to allocate energy in time is due to oxidative damage. In previous studies, after forced swimming tests ranging from 30 min to 90 min, severe oxidative stress was detected with tissue damage and markers of oxidative stress [34]. Same same with their researches, in our study, LDH and MDA were significantly higher than the normal levels by statistic compression, suggesting a state of oxidative stress. As Figure 8 depicts, antioxidant performance and oxidative stress were globally improved in the three Scp groups during exercise. Improved antioxidant performance is an overall beneficial environment for maintaining metabolic homeostasis and preventing tissular and mitochondrial damage.

On the other hand, increased mitochondrial function improved overall athletic performance, although we did not observe differences in muscle mass. Mitochondria which decide the output of ATP are the molecular layer of energy metabolism. By transforming metabolism intermediates into reduced energetic equivalents through *β*-oxidation and the TCA cycle, mitochondria serve as a crucial role in determining systemic metabolic flexibility [11]. High mitochondrial content and quality achieved by regular exercise strongly associated with muscle oxidative capacity and power and endurance performance [13]. It cannot be denied that when the size of exercise is more than a body can bear during prolonged and acute exercise, overloaded electron transport chains gives rise to mitochondrial damage [35]. There are studies proving that continuing exercise at a high intensity causes mitochondrial damages in morphology, and also after exhaustive exercise, mice exhibited mitochondrion edema, mitochondrial cristae dissolution, and degradation in SCM [34, 36]. Damaged mitochondria cause unregulated oxidative phosphorylation capacity, which impedes the ability to adjust energy materials to supply energy, reducing strength in muscle contraction. However, the function of the mitochondria during exercise depends on its own adaptability. Just as taking antioxidants for a long time does not improve endurance. Mitochondrial adaptions, as the basis of exercise adaptions, are largely dependent on their present quality and quantity and their self-regulation and upgrading capacity.

Before the swimming test, mtDNA content is increased, indicating that Scp treatment increased the overall mitochondrial quality and quantity. This result was consistent with the improved athletic ability of mice, including increased fore grip strength and endurance performance. After the swimming test, with the Scp treatment, the increased turnover of ATP indicated the improved mitochondrial capacity. According to these results, we can speculate that improved sports performance may result in increased mitochondrial ability that has been promoted before swimming and that has a direct effect on the biochemical index and mitochondrial capacity after swimming.

Secondly, the ability of the mitochondria to upgrade and self-regulate was improved by Scp treatment. Mitochondrial biogenesis involving an increase in mitochondrial number and the overall capacity of oxidative phosphorylation is one of the mechanisms of mitochondrial adaption and promotion [12]. Scp treatment significantly increased the expression pathways of PGC1*α*/NRF1 and NRF2/TFAM. Activated TFAM further regulated oxidative phosphorylation capacity and the generation of mitochondrial biogenesis proteins. However, although the improvement of oxidative stress improves the internal environment homeostasis, there is no clear connection with the improvement of mitochondrial capacity after exercise. Repair, often after muscle damage, is one of the drivers of mitochondrial biogenesis. Therefore, the present study and previous studies about improved oxidative stress cannot explain the effects of sea cucumber peptide on the increased glycogen storage and improved multitissue energy metabolism during exercise, which should be regarded as the result of overall improvement of energy metabolism homeostasis. Similarly, the increased mitochondrial quantities are also beneficial for antioxidant capacity and mean that more mitochondria can work at lower respiration for the same ATP production, producing less ROS and have stronger endogenous antioxidant capacity. Furthermore, upregulated PGC1*α* expression levels which induce mitochondrial biology positively promoted antioxidant enzyme expression. In the present study, activated PGC1*α* may be the result of the activation of AMPK and SIRT1 during exercise.

However, there are lots of studies manifesting that PGC1*α* did not express after isochronal exercise and even exhaustive swimming immediately. So from this respect and already improved mtDNA before swimming, we speculate that long-time Scp treatment promoted the occurrence of more activities. In fact, we found that Scp treatment significantly increased diet intake without increasing body weight of the mice among Scp groups, suggesting that taking sea cucumbers involved in metabolic influence resulted in improvements in exercise during feeding. This is still a conjecture. Although beyond the scope of this article, we are still working on the next step. In this article, we used multiple doses of Scp to verify their effectiveness and prevent possible errors. In the M-Scp group, we observed that blood glucose was significantly lower than that in the L-Scp and H-Scp groups. In this respect, the protein content of PGC1*α* in the M-Scp group in the liver is lower than that in the L-Scp and H-Scp groups, which is a possible cause of this phenomenon. On the other hand, we are conducting in vitro studies on myocardial and skeletal cells with sea cucumber peptide treatment, because in this study, we found that the injury markers of myocardial and skeletal muscle in Scp groups were less than those in the control group. Peptide is a mixture. Although the raw material processing method used in this article includes simulated gastrointestinal digestion, it is easy to prepare stable finished products in the industry, but it is necessary to find out the main active ingredients and do corresponding pharmacological research.

In conclusion, Scp administration increased antifatigue property by elevating mitochondrial quality and this reason could be the mechanism for the improved ability to maintain global energy homeostasis, regulate gluconeogenesis and fat catabolism, and increase antioxidant capacity. Meanwhile, the maintenance of energy homeostasis was beneficial for reducing the accumulation of metabolites.

## Figures and Tables

**Table 1:** Sequence of primers used for RT-qPCR assays.

Gene symbol	Forward primer (5′-3′)	Reverse primer (5′-3′)
*β*-Actin	GGCTGTATTCCCCTCCATCG	CCAGTTGGTAACAATGCCATGT
AMPK	CAGGCCATAAAGTGGCAGTTA	AAAAGTCTGTCGGAGTGCTGA
SIRT1	GCTGACGACTTCGACGACG	TCGGTCAACAGGAGGTTGTCT
PPAR*α*	AGAGCCCCATCTGTCCTCTC	ACTGGTAGTCTGCAAAACCAAA
PGC1*α*	TATGGAGTGACATAGAGTGTGCT	CCACTTCAATCCACCCAGAAAG
Nrf2	AGCACATCCAGACAGACACCAGT	TTCAGCGTGGCTGGGGATAT
Nrf1	AGCACGGAGTGACCCAAAC	TGTACGTGGCTACATGGACCT
TFAM	ATTCCGAAGTGTTTTTCCAGCA	TCTGAAAGTTTTGCATCTGGGT
CPT1	CTCCGCCTGAGCCATGAAG	CACCAGTGATGATGCCATTCT
PEPCK	CTGCATAACGGTCTGGACTTC	CAGCAACTGCCCGTACTCC
G6P	CGACTCGCTATCTCCAAGTGA	GTTGAACCAGTCTCCGACCA
LPL	GGGAGTTTGGCTCCAGAGTTT	TGTGTCTTCAGGGGTCCTTAG
Mt-DN2	CACGATCAACTGAAGCAGCAA	ACGATGCCAGGAGGATAATT
*β*2M	TGTCAGATATGTGCCTTCAGCAAGG	TGCTTAACTCTGCAGGCGTATG
PPAR*δ*	GAGCACACCCTTCCTTCCAG	CTCGTACTTGAGCTTCATGCG

**Table 2:** Amino acid composition of the Scp sample (g/100 g peptide).

Amino acid	Scp
Asp	6.934
Glu	9.667
Ser	3.077
His	0.635
Gly	10.757
Thr	2.582
Arg	7.916
Ala	3.831
Tyr	1.47
Cys	0.1711
Val	2.496
Met	1.108
Phe	4.681
Ile	2.207
Leu	2.78
Lys	4.954
Pro	8.868
Try	6.94 [18]

## Data Availability

The raw data used to support the findings of this study are available from the corresponding author upon request.
